# Psychological Distress Among Nurse Practitioners After the **COVID-19** Pandemic

**DOI:** 10.1097/jnr.0000000000000703

**Published:** 2025-11-10

**Authors:** Li-Chuan CHENG, Chia-Jung CHEN, Ying-Chin LIAO

**Affiliations:** 1 Department of Nursing, Dalin Tzu Chi Hospital, Buddhist Tzu Chi Medical Foundation, Chiayi, Taiwan; 2 Department of Nursing, National Cheng Kung University, Tainan, Taiwan

**Keywords:** nurse practitioners, post–COVID-19, psychological distress

## Abstract

**Background:**

Since the outbreak of the COVID-19 pandemic in 2019, frontline health care workers have experienced significant stress due to concerns about their own infection risk, fear of transmitting the virus to their families, societal stigmatization, changes in work hours, and uncertainties regarding disease progression. However, the related studies in the literature focus predominantly on psychological distress among nurses, physicians, and administrative staff during the pandemic and its factors of influence, with few studies exploring this issue in nurses during the postpandemic period. This study was developed to address this gap.

**Purpose:**

This study was designed to investigate psychological distress among nurse practitioners during the post–COVID-19 pandemic era and its associated factors.

**Methods:**

This cross-sectional study was conducted in a hospital in southern Taiwan from May 1 to July 31, 2023. The cohort included 98 nurse practitioners. Study data were collected using a personal demographics datasheet, the Depression, Anxiety, And Stress Scale-21 (DASS-21), and the stress scale of caring for highly-infectious–disease patients. Data were analyzed using descriptive statistics, the independent samples *t* test, one-way analysis of variance, Pearson’s correlation, and multiple linear regression.

**Results:**

The mean DASS-21 score was 14.17 (*SD* = 9.56), with mean scores for depression, anxiety, and stress of 8.18 (*SD* = 6.9), 6.92 (*SD* = 6.32), and 13.24 (*SD* = 8.14), respectively; all indicative of normal levels of depression, anxiety, and stress. The mean stress scale of caring for highly-infectious–disease patients was 48.89 (*SD* = 18.50). The results of a multiple linear regression analysis revealed participants aged 35–44 and older than 45 years experienced higher levels of stress while caring for highly-infectious–disease patients than their counterparts aged 25–34 years (β = 0.64, *p* = .006; β = 0.56, *p* = .027). Also, participants with 11–20 years of work experience reported lower stress in caring for highly-infectious–disease patients than their counterparts with <10 years of work experience (β = −0.46, *p* = .049).

**Conclusion/Implications for Practice:**

In this study, nurse practitioners aged 35 years and above and those with <10 years of work experience experienced greater stress following the COVID-19 pandemic. As the first study to explore psychological distress among nurse practitioners in the aftermath of the COVID-19 pandemic and its associated factors, these findings highlight the need for hospital administrators to implement support measures to support nurse practitioners tailored to age group and family status to mitigate long-term psychological distress and its impact on fatigue and care quality.

## Introduction

Severe acute respiratory syndrome coronavirus 2 (SARS-CoV-2), the causative agent of coronavirus disease 2019 (COVID-19), was first identified in December 2019 in China and rapidly spread worldwide. On January 30, 2020, COVID-19 was declared a public health emergency by the World Health Organization. This disease significantly affected economies and imposed health care burdens globally (Centers for Disease Control and Prevention, 2022; Habas et al., 2020). As of July 2023, a total of 604,674,665 COVID-19 cases and 6,507,330 deaths were confirmed worldwide, with 10,241,522 confirmed cases and 17,671 deaths reported in Taiwan (Taiwan Centers for Disease Control, 2023). This pandemic placed immense stress on frontline health care workers due to concerns about workloads, uncertainty regarding the disease, discomfort from wearing protective equipment, stress regarding infection risk, fear of transmitting the virus to family members, and other issues (Chu et al., 2021). Lasting for more than two years, the COVID-19 pandemic significantly raised the levels of stress among health care workers, which has negatively affected their mental health. Numerous studies outside of Taiwan have investigated the resulting stress, psychological distress, and related factors faced by health care professionals (Chu et al., 2021; Sinsky et al., 2021; Yamane et al., 2022).

In an exploration study on the stress experienced by health care workers (*N* = 20,655) caring for COVID-19 patients in the United States, Sinsky et al. (2021) found 48% of doctors and 63% of nurses experienced job burnout, 43% of nurses reported anxiety or depression, and 56% of nurses were burdened with excessive workloads. Burnout, fear of exposure, COVID-19-related anxiety and depression, and workload were identified as significant predictors of employee intent to resign. Hassamal et al. (2021) investigated the psychological impact of COVID-19 on health care professionals (*N* = 1,246), reporting that 21% experienced severe depression, 33% severe anxiety, and 46% significant stress. Health care professionals with 6–9 years of experience were twice as likely to experience moderate to severe depression than those with 10 years or more of experience. During the pandemic, major sources of stress included concerns about self-infection, fear of transmitting the virus to family or friends, social stigmatization, changes in work schedules, and uncertainty regarding the disease (Chu et al., 2021; Kleinpell et al., 2020).

In a study on the personal risk perceptions of health care professionals (*N* = 1,150) one year after the COVID-19 pandemic, Yamane et al. (2022) found that, during the survey period in 2021, concerns about transmitting COVID-19 to themselves and their families had significantly decreased compared with 2020. However, high stress and thoughts of resigning remained, with 57% of the health care professionals surveyed experiencing moderate to severe symptoms of posttraumatic stress disorder (PTSD) and 75% reporting moderate to high levels of job burnout. In a study of Taiwanese health care professionals caring for COVID-19 patients (*N* = 500), Lu et al. (2021) found that 15.4% experienced trauma stress syndrome, 44.6% reported insomnia symptoms, 25.6% had symptoms of depression, 30.6% experienced anxiety, and 23.4% reported stress. Kolivand et al. (2023) investigated the mental health of health care workers two years after the COVID-19 outbreak (*N* = 409), finding health care workers, especially females, continued to experience significant levels of depression (19.2%), anxiety (26.6%), and stress (18.2%). Galanis et al. (2023) investigated burnout and satisfaction among health care workers after the COVID-19 pandemic (*n* = 1,760), finding nurses to be more likely to experience moderate to high levels of burnout than other health care workers (91% vs. 79.9%, *p* < .001) and that their satisfaction was significantly lower (61% vs. 38.8%, *p* < .001). Fukushima et al. (2023) investigated the ongoing psychological effects of the pandemic on health care workers two years after the initial outbreak of COVID-19 in Japan (*N* = 719), finding that, although anxiety about infection had decreased and awareness of protection measures had increased, the psychological well-being of health care workers had continued to deteriorate. They attributed this primarily to anxiety and burnout resulting from the heavy workloads shouldered during the pandemic. Moreover, females, women in their 20s, nurses, and frontline workers were found to report higher levels of stress and burnout. Liu et al. (2023) investigated burnout and symptoms of PTSD among health care workers in China two years after the COVID-19 pandemic (*N* = 3,455), finding significant proportions (67.09% and 19.88%, respectively) experienced burnout and PTSD symptoms. Among health care workers, burnout, social support, and psychological resilience have emerged as significant predictors of PTSD symptoms.

In light of the abovementioned studies, psychological distress associated with the COVID-19 pandemic continues to affect the physical and psychological well-being and job satisfaction of health care workers, and may lead to burnout. These effects, which likely raise intent to leave and staff shortages in hospitals, are influenced by factors such as sex, age, and work environment. However, existing research has predominantly focused on nurses, physicians, and administrative staff, with limited studies investigating nurse practitioners (NPs).

Nurse practitioners, a certified subgroup of nurses, possess advanced skills and qualifications obtained through specialized training and national examinations. These nursing experts are directly involved in patient care and play a crucial role in the workforce as medical partners, assisting physicians in performing clinical duties under their guidance. As of April 2021, Taiwan had 13,851 nurse practitioner-certified nurses, with an employment rate exceeding 90% (Ministry of Health and Welfare, Taiwan, ROC, 2023). NPs have long been indispensable members of the health care team in hospitals and were particularly so during the COVID-19 pandemic. NPs bear unique responsibilities, including providing continuous patient care in isolation wards, conducting specimen collection, handling emergencies, and making critical clinical decisions in high-pressure environments. To date, limited research has focused on the mental health needs of NPs. To address this gap, the aim of this study was to investigate the psychological distress of NPs during the post–COVID-19 pandemic period and the significant factors influencing this condition. The results are expected to help guide the development of measures and policies targeting the reduction in the incidence and severity of psychological issues among nurse practitioners.

## Methods

### Study Design and Participants

This cross-sectional study recruited NPs employed at a regional 968-bed hospital in southern Taiwan who had worked as an NP during the COVID-19 pandemic. The study recruited all NPs working at the hospital ≥20 years old with a valid NP license (including 10 NPs in specialized ward, 4 NPs in specialized intensive care unit, 16 NPs in the intensive care unit, 19 NPs in medical ward, 32 NPs in surgical ward, and 17 NPs in the independent department word). The exclusion criteria were being on long-term medication for depression or anxiety and declining to participate. Data were collected from May 1 through July 31, 2023.

### Instruments

#### Demographics Datasheet

This assessment consisted of 12 items covering age, sex, educational level, marital status, number and age of children, years of nursing experience, nursing rank, direct care for COVID-19 patients, participation in COVID-19 testing, chronic disease, medication history, and religious affiliation.

#### Depression, Anxiety, and Stress Scale-21

The Depression, Anxiety, And Stress Scale-21 (DASS-21) was developed by Australian scholars Lovibond et al. (1995) by shortening the original DASS’ 42 self-assessment items to 21. The DASS-21 is suitable for use with different age groups within older adult populations and effectively assesses levels of depression, anxiety, and stress. This instrument, which is globally recognized and endorsed by medical professionals, comprises three sections of seven items each. The 21 items assess the depression (items: 3, 5, 10, 13, 16, 17, 21), anxiety (items: 2, 4, 7, 9, 15, 19, 20), and stress (items:1, 6, 8, 11, 12, 14, 18) levels of the respondent. Each item is graded on a four-point scale (0–3; 0 = *not applicable to me at all*; 3 = *applicable to me most of the time*), with a total possible score range of 0–63 and higher scores indicating a higher overall level of depression, anxiety, and stress. For each subscale, severity is categorized into five levels: normal (depression scores 0–9; anxiety scores 0–7; stress scores 0–14), mild (depression scores 10–13; anxiety scores 8–9, stress scores 15-18); Moderate (depression scores 14-20; anxiety scores 10–14; stress scores 19–25), severe (depression scores 21–27; anxiety scores 15–19; stress scores 26–33); and extremely severe (depression scores >28; anxiety scores >20; stress scores >34; Gomez, 2016).

The Chinese version of the DASS-21 was validated by Chen and Chen (2018), with Cronbach’s α values for each subscale of .87, .78 and .90, respectively. The results of confirmatory factor analysis (CFA) indicate the DASS-21 Chinese version has good factor fit, with a Comparative Fit Index (CFI) = 0.91, Tucker-Lewis Index (TLI) = 0.90, and Root Mean Square Error of Approximation (RMSEA) = 0.07. The DASS-21 is not influenced by sex, demonstrating good internal consistency.

#### Stress Scale of Caring for Patients With Highly-infectious

This questionnaire was developed by Taiwanese scholars Chuang et al. (2005) specifically for the care of patients with infectious diseases. The content was derived primarily through listening to the lived experiences of nurses who cared for patients during the severe acute respiratory syndrome (SARS) pandemic and referencing the related literature. This 32-item questionnaire comprises four sections assessing, respectively, worry and social isolation, discomfort caused by protective equipment, difficulties and anxiety related to infection control, and the workload of caring for patients. Each item is graded on a four-point scale (0–3; 0 = *not at all*; 3 = *more severe than usual*), with a total score range of 0–96 and higher scores indicating a higher level of stress. The content validity is .92 and, using the Chinese Health Questionnaire-30 (CHQ-30) to examine criterion-related validity, significant correlations (*p* < .001) were identified between the four subscales of this questionnaire and self-perceived health status in the CHQ-30. The Cronbach’s α coefficients for the four abovementioned subscales were assessed as .90, .88, .84, and .84, respectively, indicating good internal consistency.

### Data Collection and Analysis

After obtaining approval from the institutional review board under protocol number B11201008 and informed consent from participants, data were collected anonymously using coded questionnaires. This study was performed in line with the principles of the Declaration of Helsinki. Collected questionnaire data were computerized, organized, checked for completeness, and coded. Questionnaires with two or more unanswered questions were excluded from the analysis. Data were analyzed using IBM SPSS Statistics 22.0 (IBM Corp., Armonk, NY, USA). Before conducting the data analysis, considering the possibility of insufficient sample size in some groups, which may affect the maintenance of normality in the sampling distribution, the groups with insufficient numbers of individuals for each variable were merged. Subsequently, normality checks were performed on the data using the Kolmogorov-Smirnov test and the skewness and kurtosis were examined. The results indicate the data followed a normal distribution (*p* > .050), and skewness and kurtosis were within the range of ±1. Descriptive statistics (frequency, percentage, mean, and *SD*), independent sample *t* test, one-way analysis of variance, Pearson’s correlation analysis, and multiple linear regression analysis were performed according to the research objectives and the nature of the variables to elucidate the psychological distress perceptions of the participants and identify the factors significantly influencing this distress.

## Results

### Participant Characteristics

The study cohort comprised 98 nurse practitioners. The majority were 35–44 years old, female, held a university degree, were unmarried, and had no children. The majority of participants had more than 21 years of nursing experience, more than 11 years of nurse practitioner experience (NPII, 66.3%; NPIII, 18.4%), direct experience working with COVID-19 patients (89.8%), and participated in COVID-19 testing (95.9%). Most reported no chronic diseases and had never taken medicines to treat a chronic disease. The majority reported having no religious affiliation (Table 1).

**Table 1:** Participant Demographic Characteristics (*N* = 98)

Variable	*n* (%)
Age (years)	
25–34	15 (15.3)
35–44	43 (43.9)
45–54	39 (39.8)
55–64	1 (1.0)
Sex	
Female	95 (96.9)
Male	3 (3.1)
Educational level	
University degree	84 (85.7)
Master’s degree	14 (14.3)
Marital status	
Unmarried	52 (53.1)
Married	45 (45.9)
Widowed	1 (1.0)
Number of children	
0	59 (60.2)
1	10 (10.2)
2	23 (23.5)
3	5 (5.1)
4	1 (1.0)
Work experience (years)	
≤10	13 (13.2)
11–20	41 (41.8)
≥21	44 (44.9)
Licensure duration (years)	
≤5	31 (31.6)
6–10	29 (29.6)
≥11	38 (38.8)
NP rank	
NPII	65 (66.3)
NPIII	18 (18.4)
NPIV	1 (1.0)
NPV	1 (1.0)
None	13 (11.7)
Care experience	
Non-direct care	10 (10.2)
Direct care	88 (89.8)
Participation in COVID-19 testing	
No	4 (4.1)
Yes	94 (95.9)
Chronic diseases	
No	93 (94.9)
Yes	5 (5.1)
Medication history	
No	86 (87.8)
Yes	12 (12.2)
Religious affiliation	
None	37 (37.8)
Buddhism	23 (23.5)
Taoism	34 (34.7)
Christianity	1 (1.0)
Other	3 (3.1)

*Note.* NP = nurse practitioner.

### Psychological Distress and Stress Post–COVID-19 Pandemic

The DASS-21 item with the highest mean score was item 8, “I feel like I have expended a lot of mental and emotional energy” (mean = 1.39, *SD* = 0.86), followed by item 6, “I tend to overreact to certain things” (mean=0.98, *SD* = 0.76). The average overall DASS-21 score was 14.17 (*SD* = 9.56), and the mean scores for depression, anxiety, and stress were 8.18 (*SD* = 6.9), 6.92 (*SD* = 6.32), and 13.24 (*SD* = 8.14), respectively. These results suggest the participants did not experience significant negative psychological issues.

In terms of stress experienced while caring for patients with highly infectious diseases, the highest mean score was for item 13, “Wearing protective face shields and caps affects my field of vision” (mean = 2.09, *SD* = 0.77), followed by item 15, “It is inconvenient to use the restroom during working hours” (mean = 2.08, *SD* = 0.87). The average overall score for the stress scale of caring for patients with highly infectious diseases was 48.89 (*SD* = 18.50), and the average item score was 1.53. These results suggest the participants experienced a certain level of stress when caring for patients with highly infectious diseases.

### Correlations Between Participant Characteristics and, Respectively, the Depression, Anxiety, and Stress Scale-21 and Stress Scale of Caring for Patients With Highly-infectious Disease

No significant association was observed between any of the patient characteristics and the overall DASS-21 score (Table 2). Participant characteristics identified as significantly associated with perceived caregiver stress while caring for patients with highly infectious diseases included age, marital status, having children, number of children, years of work experience, and years of practitioner nursing experience (all *p* < .05). Further comparison revealed that nurse practitioners aged older than 45 years and those aged 35–44 experienced higher caregiver stress than did those do not age 25–34. The years of nurse practitioners work experience was associated with the level of caregiver stress. Those participants with over 11 years of NP experience reported higher levels of caregiver stress than their peers with <5 years of experience. Moreover, level of caregiver stress increased with number of years of work experience. However, other post hoc comparisons revealed no significant differences between the groups, with no significant associations observed between caregiver stress level and, respectively, sex, educational level, NP rank, experience caring for COVID-19 patients, participation in COVID-19 testing, chronic diseases, or religious affiliation (Table 3).

**Table 2:** Correlation Between Participant Characteristics and Overall DASS-21 Scores *(N = 98)*

Characteristic	*n*	*M*±*SD*	*F / t / r*	*p*
Age (years) ^a^			*F*=0.58	.562
25–34	15	11.73±0 7.29		
35–44	43	14.49±10.68		
≥45	40	14.75±09.07		
Sex ^b^			*t*=−0.38	.742
Female	95	14.03±09.16		
Male	3	18.67±21.22		
Educational level ^b^			*t*=0.80	.427
University degree	84	14.49±9.60		
Master’s degree	14	12.29±9.41		
Marital status ^b^			*t*=−1.98	.510
Unmarried	52	12.40±08.80		
Married/widowed	46	16.17±10.07		
Number of children ^b^			*t*=−0.33	.744
No children	59	13.92±09.04		
Have children	39	14.56±10.40		
Work experience (years) ^a^			*F*=0.02	.979
≤10	12	14.33±09.61		
11–20	41	13.95±11.40		
≥21	44	14.36±07.83		
Licensure duration (years) ^a^			*F*=0.80	.451
≤5	31	12.53±09.33		
6–10	29	15.66±10.11		
≥11	38	14.50±09.41		
NP rank ^b^			*t*=1.80	.076
NPII	65	15.52±9.84		
≥NPIII	20	11.10±8.78		
Care experience ^b^			*t*=0.11	.910
Non-direct care	10	14.50±9.70		
Direct care	88	14.14±9.59		
Participation in COVID-19 testing ^b^			*t*=0.55	.585
No	4	16.75±4.65		
Yes	94	14.06±9.71		
Chronic diseases ^b^			*t*=−0.06	.952
No	93	14.09±7.69		
Yes	5	14.22±10.43		
Medication history ^b^			*t*=−1.52	.131
No	86	13.63±9.56		
Yes	12	18.08±8.92		
Religious affiliation ^b^			*t*=−0.29	.771
None	37	13.81±10.71		
Yes	61	14.39±08.87		
Number of children ^c^			*r*=.07	.517
Work experience ^c^			*r*=.04	.704
Licensure duration ^c^			*r*=.04	.692
Chronic disease ^c^			*r*=.02	.814

*Note*. Descriptive statistics for each group are presented as mean ± *SD*. For groups with small sample sizes (e.g., male sex, the “no” group for COVID-19 testing, the “yes” group for chronic diseases), we applied both the Student’s *t* test and Welch’s *t* test to verify the outcomes. The Welch’s *t* test was used when variances were unequal. The results indicated similar outcomes for both tests, confirming the appropriateness of the Student’s *t* test as variances were equal. NP = nurse practitioner.

^a^ Independent samples *t* test. ^b^ Pearson's correlation coefficient. ^c^ One-way analysis of variance.

*p <.05. **p <.01. ***p <.001.

**Table 3:** Correlation Between Participant Characteristics and the Stress Scale of Caring for Patients With Highly Infectious Diseases *(N = 98)*

Characteristic	*n*	*M*±*SD*	*F* / *t* / *r*	*p*	Post Hoc Test
Age (years) ^a^			*F*=7.21**	.001	③>①, ②>①
① 25–34	15	33.20±18.90			
② 35–44	43	51.30±18.22			
③ ≥45	40	52.18±15.93			
Sex ^a^			*t*=0.02	.983	
Female	95	48.89±18.62			
Male	3	48.67±17.62			
Educational level ^a^			*t*=−0.44	.658	
University degree	84	48.55±18.37			
Master’s degree	14	50.93±19.91			
Marital status ^a^			*t*=2.05*	.043	
Unmarried	52	45.35±16.78			
Married/widowed	46	52.89±19.70			
Number of children ^a^			*t*=−2.77**	.007	
No children	59	44.81±16.25			
Have children	39	55.05±20.16			
Work experience (years) ^b^			*F*=4.28*	.017	NS
≤10	12	40.08±16.62			
11–20	41	45.34±19.19			
≥21	44	54.36±17.10			
Licensure duration (years) ^b^			*F*=5.02**	.008	②>①
① ≤5	31	40.63±19.91			
② 6–10	29	50.03±16.26			
③ ≥11	38	54.32±17.28			
NP rank ^a^			*t*=−1.24	.218	
NPII	65	48.89±18.96			
≥NPIII	20	54.70±15.81			
Care experience ^a^			*t*=−0.32	.749	
Non-direct care	10	47.10±18.54			
Direct care	88	49.09±18.60			
Participation in COVID-19 testing ^a^			*t*=0.92	.359	
No	4	57.25±31.02			
Yes	94	48.53±17.97			
Chronic diseases ^a^			*t*=0.04	.996	
No	93	49.00±20.18			
Yes	5	48.83±17.76			
Medication history ^a^			*t*=−0.82	.414	
No	86	48.31±19.01			
Yes	12	53.00±14.31			
Religious affiliation ^a^			*t*=−1.16	.247	
No	37	51.68±16.62			
Yes	61	47.20±19.50			
Number of children ^c^			*r*=.28**	.005	
Work experience ^c^			*r*=.34***	<.001	
Licensure duration ^c^			*r*=.28**	.005	
Chronic disease ^c^			*r*=.02	.843	

*Note.* Descriptive statistics for each group are presented as mean±*SD*. NS = not significant; NP = nurse practitioner.

^a^ Independent samples *t* test. ^b^ One-way analysis of variance. ^c^ Pearson's correlation coefficient.

*p <.05. **p <.01. ***p <.001.

### Influence of Nurse Practitioner Characteristics on the Stress Scale of Caring for Patients With Highly-Infectious Disease

A summary of the multiple linear regression analysis of the influence of nurse practitioner characteristics is presented in Table 4. Age, marital status, number of children, work experience, and licensure duration (variables that showed statistical significance in the univariate analysis) were added to the model using the “Enter” method. The *F* test for the regression model reached significance (*F* = 3.22, *p* = .003), indicating the coefficient of determination (*R^2^* = .228) to be statistically significant. This finding indicates the combination of these independent variables collectively influences the caregiver stress scale when caring for patients with high-risk infectious diseases, explaining 22.8% of the variance.

**Table 4:** Regression Analysis of Factors Influencing the Stress Scale of Caring for Highly Infectious Disease Patients

Variable	*B*	*SE*	β	*t*	*p*	VIF
Age (years)	36.83	5.06		7.28***	<.001	
35–44 vs. 25–34	24.08	8.52	0.64	2.82**	.006	5.86
≥45 vs. 25-34	21.12	9.37	0.56	2.25**	.027	6.93
Marital status (married/widowed vs. unmarried)	−2.13	5.31	−0.06	−0.40	.689	2.30
Number of children (yes vs. no)	8.61	5.44	0.23	1.58	.117	2.34
Work experience (years)						
11–20 vs. ≤10	−17.36	8.68	−0.46	−2.00*	.049	6.04
≥21 vs. ≤10	−12.83	9.88	−0.34	−1.30	.198	7.91
Licensure duration (years)						
6–10 vs. ≤5	4.09	5.44	0.10	0.75	.454	2.04
≥11 vs. ≤5	5.69	5.93	0.15	0.96	.340	2.73
Model summary						
*R* ^2^	.228					
*F*	3.22**					
*p*	.003					

*Note. B* = unstandardized regression coefficients; β = standardized regression coefficient; VIF = variance inflation.

*p <.05. **p <.01. ***p <.001.

The variance inflation (VIF) values for the independent variables ranged from 2.04 to 7.91. While some VIF values are relatively high, it should be noted that many of those are dummy variables, and multicollinearity may arise when constructing dummy variables from the same independent variable. In addition, all of the VIF values were below 10, indicating the absence of a severe multicollinearity problem among the independent variables, allowing for valid interpretation of the regression analysis results (Chiou, 2019).

In terms of *t* test results, the regression coefficient for 35–44 years old versus 25–34 years old (β = 0.64, *p* = .006) as well as for older than 45 years old versus 25–34 years old (β = 0.56, *p* = .027) were significantly positive, suggesting that nurse practitioners aged 35 and older experience higher caregiver stress when working with high-risk infectious disease patients than their peers aged 25–34 years. Furthermore, in terms of years of work experience, the regression coefficient for “11–20 years versus <10 years” was significantly negative (β = −0.46, *p* = .049), indicating NPs who have worked for 11–20 years perceive lower stress in caring for high-risk infectious disease cases than those with <10 years of work experience. No other variables were found to independently affect this stress scale (Table 4).

## Discussion

This study investigated the results of the DASS-21 on nurse practitioners in the postpandemic era and identified relevant factors that influence the stress scale of caring for highly-infectious–disease patients. The findings indicate nurse practitioners caring for COVID-19 patients experience mild psychological distress, with notable indicators including feelings of mental and emotional fatigue, tendencies to overreact to certain situations, and difficulties in finding relaxation. The stress of caring for patients with highly infectious diseases was significantly influenced by factors such as the discomfort caused by protective gear, which hindered visibility, made restroom access challenging, and created barriers to effective communication. In addition, concerns about potentially transmitting the virus to family members were prominent sources of anxiety for these health care professionals.

The findings of this study align with Galanis (2023), which examined burnout and satisfaction among health care professionals in the postpandemic era. Despite reduced workload and intensity, health care professionals continue to face psychological distress attributable to pandemic-related fears such as infection risk, transmitting the virus to family, staff shortages, and insufficient support, leading to heightened burnout. Even as the pandemic subsides, its psychological impact persists among health care professionals.

NP demographic characteristics were not found to relate significantly to depression, anxiety, or stress. This finding contrasts with the many studies reporting sex, age, marital status, years of work experience, and having children to have significant associations with depression and anxiety (Hassamal et al., 2021; Sinsky et al., 2021; Sirois & Owens, 2021). This difference in findings may be attributable to the extensive training programs and response measures undertaken at the study hospital at the peak of the pandemic. The measures included establishing a command center; holding daily discussions on the condition of COVID-19 patients; monitoring the availability of protective equipment and providing emergency allocations when shortages occurred; providing accommodations to alleviate concerns about bringing the virus home; offering meals, fruit, and traditional Chinese preventive tea; and reducing nurse-to-patient ratios to ensure health care workers had the physical strength to care for patients. The findings of previous studies suggest organizational support, sufficient information on infectious diseases, relevant protective training, adequate protective equipment, and adequate staffing can significantly reduce psychological distress (Chen et al., 2006; Chigwedere et al., 2021; Jung et al., 2020).

Age, marital status, number of children, years of work experience, and licensure duration were found to relate significantly to caregiver stress. Specifically, participants aged 35 and above earned higher scores on the stress scale than their younger peers, and nurse practitioners with 11–20 years of work experience reported lower stress in caring for high-risk infectious disease cases than their peers with 10 or less years of work experience. None of the other demographic variables considered were shown to significantly affect stress. These findings are consistent with previous findings that older health care workers feel more-severe psychological distress and stress than their younger colleagues (Han et al., 2020; Nie et al., 2020). Prior research indicates that stress correlates negatively with years of work experience; thus, health care personnel with extensive experience generally report lower levels of caregiving stress, pressure, and anxiety than their less-experienced peers (Hassamal et al., 2021; Kim et al., 2021). This concurs with the finding in this study that participants with 11–20 years of work experience report significantly lower caregiving stress in caring for patients with high-risk infectious diseases compared with their less-experienced peers. Notably, no significant difference in caregiving stress was observed between those with over 21 years of work experience and those with <10 years of work experience. Other factors outside the scope of this research may have contributed to this inconsistency in the results. In summary, the findings indicate that certain demographic characteristics influence the level of stress perceived by nurse practitioners when caring for patients with highly infectious diseases.

Future research on this topic should include qualitative studies to further elucidate the experiences, perspectives, and feelings of nurse practitioners when caring for high-risk infectious patients. This may inspire further research to investigate how different personal attributes affect the psychological distress perceptions and coping strategies of health care workers under various situations.

### Limitations

This study was affected by several limitations. First, the study cohort included only nurse practitioners working at a single hospital, which may limit the generalizability of the findings. Future research may recruit nurse practitioners from different health care settings to improve the representativeness of the results. Second, this study focused on the psychological distress experienced by nurse practitioners caring for patients with specific infectious diseases. However, psychological distress may evolve over time or vary in different situations. Because the study data were collected during a relatively stable phase of the COVID-19 pandemic, the study results may underestimate the true extent of psychological distress experienced.

### Conclusions and Recommendations

The findings of this cross-sectional study suggest elevated levels of psychological distress may persist well beyond the peak of a pandemic. This study was the first to investigate psychological distress and its associated factors among nurse practitioners in the post-COVID-19 pandemic era. Understanding and addressing this distress will be crucial to preventing long-term negative consequences such as burnout and subpar patient care quality. This study provides valuable insights for hospital administrators that increase their understanding and appreciation of the psychological challenges faced by nurse practitioners in the postpandemic era and guide their enactment of early preventive/remedial measures. These measures may include providing mental health support during exceptional epidemic situations and tailoring support strategies to accommodate nurses of different ages and family situations, which, in turn, can help reduce the risk of burnout and sustain the provision of high-quality patient care.
